# The role of senior researchers in promoting good science: Obstacles and enablers

**DOI:** 10.12688/openreseurope.19140.1

**Published:** 2025-01-23

**Authors:** Susanne van den Hooff, Signe Mežinska

**Affiliations:** 1 Care Ethics, University of Humanistic Studies, Kromme Nieuwegracht 29, Utrecht, 3512, The Netherlands; 2 Institute of Clinical and Preventive Medicine, Latvijas Universitate Faculty of Medicine and Life Sciences, Jelgavas Str. 3, Riga, LV-1004, Latvia

**Keywords:** research ethics, research integrity, research misconduct, research culture, senior researchers, slow science

## Abstract

This essay examines senior researchers’ professional responsibilities in fostering ethical research practices within their teams, as outlined in the ALLEA European Code of Conduct for Research Integrity. Senior researchers have an important role in preventing research misconduct and promoting a supportive academic environment. However, pressures within academia - particularly the ‘publish or perish’ culture - can lead to stress and potentially unethical practices, including power misuse, exploitation, and neglect of supervisory responsibilities. This essay explores the challenges senior researchers face in fulfilling ethical responsibilities and highlights a ‘slow science’ approach and targeted training to prioritize quality over quantity and to promote better mentorship practices.

## Introduction

Senior researchers, like all researchers, bear the responsibility of upholding research integrity in their work. Nevertheless, they also carry significant responsibility for ensuring that their research group engages in good scientific practices and for taking necessary steps to prevent research misconduct and unacceptable research practices. In this article, we define senior researchers as those who oversee a research group. Senior researchers are tasked with creating a positive and supportive research environment through implementing effective supervision, offering clear guidance and serving as ethical role models ( Pizzolato & Dierickx, 2023).

Several recent cases demonstrate that senior researchers are ultimately held accountable for the misconduct or unacceptable practices of colleagues under their supervision, e.g., case of Stanford president's resignation ( Kozlov, 2023).

In 2024, a high-profile case in the Netherlands drew widespread media attention to a professor accused of gross negligence. The case pertains to the article discussing excess mortality across Western countries since the COVID-19 pandemic ( Mostert *et al.*, 2024). This article first-authored by a postdoctoral researcher raised doubts about the safety of COVID-19 vaccination, asserting that the ongoing excess mortality observed since the pandemic was caused by COVID-19 vaccines. The supervisor of the postdoctoral researcher, who was a co-author of the manuscript, expressed concerns about the first draft of the paper and even stated that it would be better if he did not associate his name with it, as showed by the reconstruction of the case in a Dutch newspaper ‘De Volkskrant’:

“In May 2023 the supervisor showed his hesitation towards the article. "Still food for thought," is the brief response he sends back four days later. He added comments in the margins of the draft text, such as: "I really can't imagine that there are no reliable figures on the COVID vaccines. You need to look these up and cite them." Further down: "I find this difficult, as it suggests that many people have died due to the vaccines. Also, there is nothing about the protection offered by vaccines." He even deems it "unscientific". Perhaps it would be better if he did not associate his name with it, he suggests. "What I miss is an explanation of why we are writing about this from our affiliations." And later: "What I also miss is something about children, and childhood cancer. You and I are paid for that. (...) If that’s not included, I don’t want to be a co-author, and I think you should write this on a personal basis." ( Keulemans, 2024)

Nevertheless, the paper was published with the supervisor being included as one of the co-authors. The research paper garnered significant media coverage, including reports from The Telegraph, New York Post, and Berliner Zeitung, and was shared extensively on social media. At the same time, the paper sparked substantial academic debate. The Princess Maxima Center listed as the affiliation of three of the authors, started an research integrity investigation, and the journal issued an expression of concern ( BMJ, 2024). The postdoc who authored the paper resigned. While the Scientific Integrity Committee of the Princess Máxima Center did not conclude that the senior researcher violated scientific integrity, it criticized him for gross negligence. After receiving a revised version of the paper in which his feedback had not been addressed, the senior researcher failed to ensure that his comments were properly incorporated. ( Keulemans, 2024)

This and similar cases underscore the serious consequences senior researchers may face due to insufficient supervision and challenge the common assumption that senior researchers are invulnerable due to their institutional position and authority.

The ALLEA European Code of Conduct for Research Integrity ( All European Academies, 2023) providing an EU wide framework for research integrity includes several professional responsibilities of senior researchers. Section 2.2 of the ALLEA code “Training, supervision and mentoring” mentions that *“senior researchers, research leaders, and supervisors mentor their team members, lead by example, and offer specific guidance and training to properly develop and structure their research activities”* and *“researchers across the entire career path, from early career to most senior level, undertake training in ethics and research integrity”* (p.6). At the same time *“Misusing seniority to encourage violations of research integrity or to advance one's own career”* is included in ALLEA code as an example of research misconduct.

In this essay we seek to address how senior researchers are implementing the professional responsibilities outlined in the ALLEA code in their daily practice. We conclude this essay by sharing insights on how to support senior researchers in becoming good supervisors, team managers, and fulfil their responsibilities in an ethical manner.

## Responsibilities of senior researchers

To uphold their ethical responsibilities to mentor, guide, and train research teams, senior researchers must recognise and understand their responsibilities outlined in the ALLEA code, especially their implementation in the context of academic culture of “publish or perish”. This culture values frequent publishing in high-impact journals as a key measure of a researcher’s success. As a result, there is a risk that researchers may prioritise the quantity of publications over the quality of research, potentially leading to research misconduct, unacceptable practices or extreme publishing behaviour. ( Ioannidis *et al.*, 2024) 

A qualitative study by Wäscher *et al.* (2020) explored what senior researchers themselves see as their professional academic responsibilities, how they assume them, and what challenges they perceive with respect to their responsibilities. Their results showed that senior researchers hold themselves responsible for doing good science, which was described by one researcher as “First of all, to do good science, being honest, being critical. The foundation is being self-critical and then start questioning (IP12)” ( Wäscher *et al.*, 2020).

Senior researchers are responsible for preventing unacceptable practices and research misconduct in their team. In a study by Glerup *et al.* (2017) senior researchers acknowledged these responsibilities and described them as a part of their daily scientific practice for producing good science, such as taking care of social and ethical challenges, doing research in meticulous ways, taking care of the people in their research group, managing professional relationships within and beyond the group for all, securing funding (ongoing collective work of writing grant applications and political lobbying for their field of research), creating ‘impact’, and producing scientific results that are legitimate in the eyes of the public by participation in public debate about developments related to their own discipline or technological area ( Glerup *et al.*, 2017).

These professional responsibilities of senior researchers together with the academic pressure to produce scientific results can lead to high levels of stress for them. Interviewees in a study by Aubert Bonn & Pinxten (2021) described the pressure to perform, especially to deliver research results, as well as the academic culture of ‘publish or perish’ as significant issues when asked about problems affecting the culture of science and threatening integrity ( Aubert Bonn & Pinxten, 2021). Conesa Carpintero & González Ramos (2018) interviewed academics who said that they devote more hours to work than established in their labour contracts. They have to work in a “high-pressure environment, abuse in power relations and lack tools and support (..)”, that “eventually led to feelings of isolation and anxiety that produced a lack of selfcare and emotional dependence on giving results to their boss (..)”( Conesa Carpintero & González Ramos, 2018). It seems that it is often difficult for senior researchers to balance between the different tasks of their professional responsibility in team management - ensuring good scientific practices and responding to the pressures to produce scientific results, such as publications. At the same time, research shows that senior researchers lack rewards and recognition for their work in ensuring research integrity, as well as specific training ( Pizzolato & Dierickx, 2023).

## Pressure to perform and the risk of power misuse

The high level of pressure to perform in some cases may lead to the situation where senior researchers misuse their seniority to pressure their team to perform so that the academic goals that are set are achieved. Misuse of power in academic settings manifests in several potentially harmful ways, particularly affecting early career researchers. One common issue is the exploitation of labour ( Conesa Carpintero & González Ramos, 2018). Senior researchers, who hold significant authority, may assign excessive workloads to early career researchers, expecting them to perform tasks well beyond their responsibilities, such as administrative duties. This exploitation is often exacerbated by the pressure early career researchers feel to comply in order to secure future recommendations, publications, or career opportunities.

Horbach *et al.* (2020) results showed that early career researchers did not report misconduct due to their fear of negative consequences, such as losing future opportunities or hampering their social relations at work, the distrust of the management’s willingness to take any corrective action, and the belief that reporting would not lead to any changes, notably due to certain individuals being protected. These responses showed “how seniority, hierarchy and power issues affected respondents’ decision not to report an alleged case” (p. 1604) and that misuse of seniority may actually be one of the causes of misconduct ( Horbach *et al.*, 2020).

Another example of misuse of seniority is unjustified appropriation of intellectual credit. In some cases, senior researchers may claim sole or disproportionate credit for the work and ideas of their early career colleagues, even when the early career researchers have made significant contributions to a research project. This can severely hinder the professional development of early career researchers, as their names may not be adequately featured in publications or grant applications, which are critical for academic advancement. For example, the qualitative data from a study on misuse of co-authorship in medical theses in Sweden by Helgesson *et al.* (2018) highlighted misuse of power by senior researchers and several reasons why authorship is often not handled in line with authorship guidelines. Respondents suggested that some senior researchers disregard these guidelines to ensure that their own or other colleagues' names appear on as many articles as possible, by adding co-authors without meaningful contribution. Doctoral students often find it difficult to whistle blow these practices, sometimes facing negative consequences when they do ( Helgesson *et al.*, 2018).

The misuse of seniority and power might play a role in the prevalence of research misconduct amongst early career researchers and a higher prevalence of unacceptable research practices. A Dutch survey among academic researchers showed that due to insufficient supervision or mentoring, early career researchers and PhD candidates engage more often in any frequent unacceptable research practices than other academic ranks ( Gopalakrishna *et al.*, 2022). Another Dutch study of academics postulated that this may be in part explained by the consistent lack of good supervision and mentoring of early career researchers ( Haven *et al.*, 2019).

## Conclusions

How can the academic community and policy makers support senior researchers to be good supervisors, team managers and to ethically fulfil their responsibilities?

First, a slow science approach with a focus on quality over quantity in research might help senior researchers to allocate more time for supervision and mentoring their research team. Stengers (2018) provided us with some ideas that might be useful for changing the prevailing research culture. She states that slow science should reject ‘fast’ accountability, blindly imposed by the management. ( Stengers, 2018) The ever-growing publication pressure is influencing the academic world. To go against the hectic publication pressure “the assessment of research, researchers and research organisations should recognise the diverse outputs, practices and activities that maximise the quality and impact of research”, which is the vision of the Coalition for Advancing Research Assessment ( CoARA, 2022).

Second, more training courses specifically tailored to the unique challenges and responsibilities of senior researchers could meaningfully support them in their roles. Such courses might cover advanced mentorship strategies, balancing pressure to perform and conflict resolution, ethical decision-making, and practical approaches to supervising diverse research teams. While some senior researchers may naturally excel in supervising and promoting good scientific practice, many may benefit from targeted training. Institutions and national level research integrity bodies should prioritize developing training programs to senior researchers for ensuring responsible research practices.

Care and consideration should not only be given to early career researchers who need support but also to senior researchers who could be helped by a slow science approach and tailored training.

## Ethics and consent

Ethics and consent were not required.

## Data Availability

No data are associated with this article.

## References

[ref-1] All European Academies A: The European code of conduct for research integrity. All European Academies (ALLEA),2023; Retrieved November 8, 2024. Reference Source

[ref-2] Aubert Bonn N Pinxten W : Rethinking success, integrity, and culture in research (part 2)—a multi-actor qualitative study on problems of science. *Res Integr Peer Rev.* 2021;6(1): 3. 10.1186/s41073-020-00105-z 33441167 PMC7807493

[ref-3] BMJ: Expression of concern: excess mortality across countries in the western world since the COVID-19 pandemic: 'Our World in Data' estimates of January 2020 to December 2022. *BMJ Public Health.* 2024;2(1): e000282eoc. 10.1136/bmjph-2023-000282eoc PMC13482250 42614536

[ref-4] CoARA: Agreement on reforming research assessment.2022; Retrieved November 9, 2024. Reference Source

[ref-5] Conesa Carpintero E González Ramos AM : Accelerated researchers: psychosocial risks in gendered institutions in academia. *Front Psychol.* 2018;9:1077. 10.3389/fpsyg.2018.01077 30072928 PMC6062159

[ref-6] Glerup C Davies SR Horst M : ‘Nothing really responsible goes on here’: scientists’ experience and practice of responsibility. *J Responsible Innov.* 2017;4(3):319–336. 10.1080/23299460.2017.1378462

[ref-7] Gopalakrishna G Ter Riet G Vink G : Prevalence of questionable research practices, research misconduct and their potential explanatory factors: a survey among academic researchers in the Netherlands. *PLoS One.* 2022;17(2): e0263023. 10.1371/journal.pone.0263023 35171921 PMC8849616

[ref-8] Haven TL Tijdink JK Martinson BC : Perceptions of research integrity climate differ between academic ranks and disciplinary fields: results from a survey among academic researchers in Amsterdam. *PLoS One.* 2019;14(1): e0210599. 10.1371/journal.pone.0210599 30657778 PMC6338411

[ref-9] Helgesson G Juth N Schneider J : Misuse of coauthorship in medical theses in Sweden. *J Empir Res Hum Res Ethics.* 2018;13(4):402–411. 10.1177/1556264618784206 29985088

[ref-10] Horbach SP Breit E Halffman W : On the willingness to report and the consequences of reporting research misconduct: the role of power relations. *Sci Eng Ethics.* 2020;26(3):1595–1623. 10.1007/s11948-020-00202-8 32103454 PMC7286863

[ref-11] Ioannidis JP Collins TA Baas J : Evolving patterns of extreme publishing behavior across science. *Scientometrics.* 2024;129:5783–5796. 10.1101/2023.11.23.568476

[ref-12] Keulemans M : Wereldwijde blunder van het prinses Máxima centrum: opeens verspreidde het corona-desinformatie. Wat ging er mis? *de Volkskrant.* 2024; 26 November. Reference Source

[ref-13] Kozlov M : What the stanford president's resignation can teach lab leaders. *Nature.* 2023. 10.1038/d41586-023-02438-3 37501000

[ref-14] Mostert S Hoogland M Huibers M : Excess mortality across countries in the Western World since the COVID-19 pandemic: â Our World in Dataâ estimates of January 2020 to December 2022. *BMJ Public Health.* 2024;2(1): e000282. 10.1136/bmjph-2023-000282 PMC13482250 42614536

[ref-15] Pizzolato D Dierickx K : The mentor’s role in fostering research integrity standards among new generations of researchers: a review of empirical studies. *Sci Eng Ethics.* 2023;29(3): 19. 10.1007/s11948-023-00439-z 37160826

[ref-16] Stengers I : Another science is possible: a manifesto for slow science. (S. Muecke, Trans.). Polity Press,2018. Reference Source

[ref-17] Wäscher S Biller-Andorno N Deplazes-Zemp A : “I don’t want to do anything bad.” Perspectives on scientific responsibility: results from a qualitative interview study with senior scientists. *Nanoethics.* 2020;14(2):135–153. 10.1007/s11569-020-00365-5

