# The role of senior researchers in promoting good science: Obstacles and enablers

**DOI:** 10.12688/openreseurope.19140.2

**Published:** 2025-04-24

**Authors:** Susanne van den Hooff, Signe Mežinska

**Affiliations:** 1 Care Ethics, University of Humanistic Studies, Kromme Nieuwegracht 29, Utrecht, 3512, The Netherlands; 2 Institute of Clinical and Preventive Medicine, Latvijas Universitate Faculty of Medicine and Life Sciences, Jelgavas Str. 3, Riga, LV-1004, Latvia

**Keywords:** research ethics, research integrity, research misconduct, research culture, senior researchers, slow science

## Abstract

This essay examines senior researchers’ professional responsibilities in fostering ethical research practices within their teams, as outlined in the ALLEA European Code of Conduct for Research Integrity. Senior researchers have an important role in preventing research misconduct and promoting a supportive academic environment. However, pressures within academia - particularly the ‘publish or perish’ culture - can lead to stress and potentially unethical practices, including power misuse, exploitation, and neglect of supervisory responsibilities. This essay explores the challenges senior researchers face in fulfilling their responsibilities and highlights a ‘slow science’ approach and targeted training to prioritize quality over quantity and to promote better supervision practices.

## Introduction

Senior researchers, like all researchers, bear the responsibility of upholding research integrity in their work. Nevertheless, they also carry significant responsibility for ensuring that their research group engages in good scientific practices and for taking necessary steps to prevent research misconduct and unacceptable research practices. In this article, we define senior researchers as those who have extensive experience and expertise in their field of research and who supervise a research group. The use of the term ‘senior researcher’ can vary depending on the scientific field and country. Nevertheless, despite these differences, it is still possible and meaningful to broadly reflect on the role of senior scientists in fostering research integrity, as exemplified by the ALLEA European Code of Conduct for Research Integrity ( All European Academies, 2023). In general, senior researchers are tasked with creating a positive and supportive research environment through implementing effective supervision, offering clear guidance and serving as ethical role models ( Pizzolato & Dierickx, 2023). Compared to mentoring, which offers broad and informal support, supervision is a more task-oriented and formal activity, focusing on managing specific responsibilities and ensuring tasks are completed responsibly and efficiently.

The ALLEA European Code of Conduct for Research Integrity ( All European Academies, 2023) providing an EU wide framework for research integrity outlines several professional responsibilities of senior researchers. Section 2.2 of the ALLEA code “Training, supervision and mentoring” mentions that *“senior researchers, research leaders, and supervisors mentor their team members, lead by example, and offer specific guidance and training to properly develop and structure their research activities”* and *“researchers across the entire career path, from early career to most senior level, undertake training in ethics and research integrity”* (p.6). Additionally, the ALLEA code identifies *“Misusing seniority to encourage violations of research integrity or to advance one's own career”* as an example of research misconduct.

The consequences of not adhering to these ethical standards are illustrated by recent cases where senior researchers have been held accountable for the misconduct or unacceptable practices of colleagues under their supervision. For example, the resignation of Stanford’s president ( Kozlov, 2023) illustrates the serious consequences of failing to foster research integrity and motivate the laboratory staff supervised by a senior researcher to work with integrity.

In 2024, a high-profile case in the Netherlands drew widespread media attention to a professor accused of gross negligence. The case pertains to the article discussing excess mortality across Western countries since the COVID-19 pandemic ( Mostert *et al.*, 2024). This article first-authored by a postdoctoral researcher raised doubts about the safety of COVID-19 vaccination, asserting that the ongoing excess mortality observed since the pandemic was caused by COVID-19 vaccines. The supervisor of the postdoctoral researcher, who was a co-author of the manuscript, expressed concerns about the first draft of the paper and even stated that it would be better if he did not associate his name with it, as showed by the description of the case in a Dutch newspaper ‘De Volkskrant’:

“In May 2023 the supervisor showed his hesitation towards the article. "Still food for thought" is the brief response he sends back four days later. He added comments in the margins of the draft text, such as: "I really can't imagine that there are no reliable figures on the COVID vaccines. You need to look these up and cite them." Further down: "I find this difficult, as it suggests that many people have died due to the vaccines. Also, there is nothing about the protection offered by vaccines." He even deems it "unscientific". Perhaps it would be better if he did not associate his name with it, he suggests. "What I miss is an explanation of why we are writing about this from our affiliations." And later: "What I also miss is something about children, and childhood cancer. You and I are paid for that. (...) If that’s not included, I don’t want to be a co-author, and I think you should write this on a personal basis." ( Keulemans, 2024)

Nevertheless, the paper was published with the supervisor being included as one of the co-authors. The research paper garnered significant media coverage, including reports from The Telegraph, New York Post, and Berliner Zeitung, and was shared extensively on social media. At the same time, the paper sparked substantial academic debate. The Princess Maxima Center listed as the affiliation of three of the authors, started a research integrity investigation, and the journal issued an expression of concern ( BMJ, 2024). The postdoc who authored the paper resigned. While the Scientific Integrity Committee of the Princess Maxima Center did not conclude that the senior researcher violated scientific integrity, it criticized him for gross negligence. After receiving a revised version of the paper in which his feedback had not been addressed, the senior researcher as a supervisor failed to ensure that the article met scientific standards and was based on facts. Despite this, he did not object to remaining listed as a co-author. He also failed to review later versions containing feedback from the journal. ( Keulemans, 2024)

This and similar cases ( Caron *et al.*, 2025) underscore the serious consequences senior researchers may face due to insufficient supervision and challenge the common assumption that senior researchers are invulnerable due to their institutional position and authority. Supervision is inherently subject to scrutiny and accountability in academia. Research misconduct cases often involves supervisors who did not perform the research themselves but are responsible for overseeing the work, and the responsibility of senior researchers in these cases may be evaluated based on the concept of recklessness. ( Caron *et al.*, 2025) Both the early career researcher and the supervisor may be held accountable for research misconduct or unacceptable practices carried out by the supervisee. Also, accusations of power abuse can severely harm the reputation and career of senior researchers. ( Lasser *et al.*, 2021) Senior researchers are indeed responsible for high-quality supervision; nevertheless, the pressure that supervisors may experience when they are overwhelmed by their workload and unable to dedicate sufficient time to effectively oversee their teams can lead to decreased quality of supervision, increased risk of recklessness, and potential issues with research integrity. This is not merely an individual problem for senior researchers; it must be addressed as a systemic issue and a matter of academic culture. Institutions need to implement policies and practices that ensure adequate support and time for supervision to maintain high standards of research integrity.

In this essay we seek to address how senior researchers are expected to implement the professional responsibilities outlined in the ALLEA code in their daily practice. We conclude this essay by sharing insights on how to support senior researchers in becoming good supervisors, team managers, and fulfil their responsibilities in an ethical manner.

## Responsibilities of senior researchers

To uphold their responsibilities to supervise, guide, and train research teams, senior researchers must recognise and understand their obligations outlined in the ALLEA code, particularly their implementation in the context of demanding academic culture of “publish or perish”. This culture values frequent publishing in high-impact journals as a key measure of a researcher’s success. As a result, there is a risk that researchers may prioritise the quantity of publications over the quality of research and supervision, potentially leading to research misconduct, unacceptable practices or extreme publishing behaviour. ( Ioannidis *et al.*, 2024) 

A qualitative study by Wäscher *et al.* (2020) explored what senior researchers themselves see as their professional academic responsibilities, how they assume them, and what challenges they perceive with respect to their responsibilities. Their results showed that senior researchers hold themselves responsible for doing good science, which was described by one researcher as “First of all, to do good science, being honest, being critical. The foundation is being self-critical and then start questioning (IP12)” ( Wäscher *et al.*, 2020).

Senior researchers are responsible for preventing unacceptable practices and research misconduct in their team. In a study by Glerup *et al.* (2017) senior researchers acknowledged these responsibilities and described them as a part of their daily scientific practice for producing good science, such as taking care of social and ethical challenges, doing research in meticulous ways, taking care of the people in their research group, managing professional relationships within and beyond the group for all, securing funding (ongoing collective work of writing grant applications and political lobbying for their field of research), creating ‘impact’, and producing scientific results that are legitimate in the eyes of the public by participation in public debate about developments related to their own discipline or technological area ( Glerup *et al.*, 2017).

From a different perspective, Conesa Carpintero & González Ramos (2018) interviewed academics who said that they devote more hours to work than established in their labour contracts. They have to work in a “high-pressure environment, abuse in power relations and lack tools and support (..)”, that “eventually led to feelings of isolation and anxiety that produced a lack of selfcare and emotional dependence on giving results to their boss (..)”( Conesa Carpintero & González Ramos, 2018). It seems that it is often difficult for senior researchers to balance between the different tasks of their professional responsibility in team management - ensuring good scientific practices and responding to the pressures to produce scientific results, such as publications. At the same time, research shows that senior researchers lack rewards and recognition for their work in ensuring research integrity, as well as specific training ( Pizzolato & Dierickx, 2023).

## Pressure to perform and the risk of power misuse

The high level of pressure to perform in some cases may lead to the situation where senior researchers misuse their seniority to pressure their team to perform so that the academic goals that are set are achieved. Misuse of power in academic settings manifests in several potentially harmful ways, particularly affecting early career researchers. One common issue is the exploitation of labour ( Conesa Carpintero & González Ramos, 2018). Senior researchers, who hold significant authority, may assign excessive workloads to early career researchers, expecting them to perform tasks well beyond their responsibilities, such as administrative duties. This exploitation is often exacerbated by the pressure early career researchers feel to comply in order to secure future recommendations, publications, or career opportunities.

Horbach *et al.* (2020) results showed that early career researchers did not report misconduct due to their fear of negative consequences, such as losing future opportunities or hampering their social relations at work, the distrust of the management’s willingness to take any corrective action, and the belief that reporting would not lead to any changes, notably due to certain individuals being protected. These responses showed “how seniority, hierarchy and power issues affected respondents’ decision not to report an alleged case” (p. 1604) and that misuse of seniority may actually be one of the causes of misconduct ( Horbach *et al.*, 2020).

Another example of misuse of seniority is unjustified appropriation of intellectual credit. In some cases, senior researchers may claim sole or disproportionate credit for the work and ideas of their early career colleagues, even when the early career researchers have made significant contributions to a research project. This can severely hinder the professional development of early career researchers, as their names may not be adequately featured in publications or grant applications, which are critical for academic advancement. For example, the qualitative data from a study on misuse of co-authorship in medical theses in Sweden by Helgesson *et al.* (2018) highlighted misuse of power by senior researchers and several reasons why authorship is often not handled in line with authorship guidelines. Respondents suggested that some senior researchers disregard these guidelines to ensure that their own or other colleagues' names appear on as many articles as possible, by adding co-authors without meaningful contribution. Doctoral students often find it difficult to whistle blow these practices, sometimes facing negative consequences when they do ( Helgesson *et al.*, 2018).

The misuse of seniority and power might play a role in the prevalence of research misconduct amongst early career researchers and a higher incidence of unacceptable research practices. A Dutch survey among academic researchers showed that, due to insufficient supervision, early career researchers and PhD candidates engage more frequently in unacceptable research practices than other academic ranks ( Gopalakrishna *et al.*, 2022). Another Dutch study of academics postulated that this may be in part explained by the consistent lack of good supervision and mentoring of early career researchers ( Haven *et al.*, 2019).

## Discussion and conclusions

How can the academic community and policy makers support senior researchers in becoming good supervisors, ethically responsible team managers and trustworthy leaders?

First, adopting a slow science approach with a focus on quality over quantity in research could enable senior researchers to allocate more time for supervision their research teams. Stengers (2018) offers valuable insights for transforming the current research culture. She states that slow science should reject ‘fast’ accountability, blindly imposed by the management. ( Stengers, 2018) The ever-growing publication pressure is impacting the academic world. To counter the hectic publication pressure “the assessment of research, researchers and research organisations should recognise the diverse outputs, practices and activities that maximise the quality and impact of research”, which is the vision of the Coalition for Advancing Research Assessment ( CoARA, 2022).

An example of a novel approach to rewarding academics, including the recognition of supervision and leadership roles, can be seen in the Dutch Recognition & Rewards programme. This programme shifts the focus from a one-sided emphasis on research performance, which often results in excessive pressure to generate fast results, to rewarding other qualities such as leadership, impact, and collaboration. ( Lagendijk & Wiering, 2024) It encourages good academic leadership at all levels, from early career researchers to senior academics and aims at promoting a culture of thoroughness, ensuring that supervised research meets high standards of quality and integrity. By reducing pressure and the emphasis on speed, senior researchers could focus more on fostering long-term growth and development within their teams.

Second, more training courses specifically tailored to the unique challenges and responsibilities of senior researchers could meaningfully support them in their roles. ( Pizzolato & Dierickx, 2023) Such courses might focus on advanced supervising strategies, balancing pressure to perform with high-quality science, strengthening conflict resolution skills, fostering ethical decision-making, and suggesting practical approaches for supervising diverse research teams. While some senior researchers may naturally excel in supervising and promoting good scientific practice, many may benefit from targeted training. Institutions and national level research integrity bodies should prioritize developing training programs to senior researchers for ensuring responsible research practices.

Useful materials and guidelines for development and implementation of training courses for senior researchers are recently compiled by several Horizon projects, e.g., SOPs4RI and BEYOND. The SOPs4RI project provides guidance to research institutions on developing research integrity education strategies for senior researchers by emphasizing the importance of education and training to equip them with tools to promote responsible practices. The guidelines are designed to be flexible and adaptable, catering to the diverse needs of various research institutions. ( Labib *et al.*, 2023) The BEYOND project focuses on enhancing supervision skills that can help senior researchers more effectively guide early career researchers and foster a culture of integrity within their teams. ( Pizzolato *et al.*, 2024) By incorporating these resources into training courses, institutions and organizations can promote a more balanced and supportive environment that values both individual and team achievements.

Furthermore, a valuable tool that can be used for training senior researchers and fostering a culture of research integrity is reverse mentoring. Pizzolato and Dierickx (2022) have suggested this novel approach, where early career researchers mentor senior colleagues to bridge generational gaps and promote continuous learning. ( Pizzolato & Dierickx, 2022) They suggest that reverse mentoring can improve intergenerational relationships and build a culture of transparency and trust, as well as support the professional development of both early career and senior researchers. 

In conclusion we would like to emphasize that care and consideration should not only be given to early career researchers who need support but also to senior researchers who could be helped by a slow science approach and tailored training. While senior researchers hold the responsibility for providing high-quality supervision, the pressures they face in the academic environment can compromise its effectiveness. Addressing this issue requires a systemic approach and a shift in academic culture. Institutions must implement policies and practices that offer adequate support, ensure sufficient time, and reward supervision, thereby supporting high standards of research integrity.

## Ethics and consent

Ethics and consent were not required.

## Data Availability

No data are associated with this article.

## References

[ref-1] All European Academies: The European code of conduct for research integrity. All European Academies (ALLEA),2023; Retrieved November 8, 2024. Reference Source

[ref-3] BMJ: Expression of concern: excess mortality across countries in the western world since the COVID-19 pandemic: 'Our World in Data' estimates of January 2020 to December 2022. *BMJ Public Health.* 2024;2(1): e000282eoc. 10.1136/bmjph-2023-000282eoc PMC13482250 42614536

[ref-18] Caron MM Dohan SB Barnes M : Defining “recklessness” in research misconduct proceedings. *Account Res.* 2025;32(2):120–142. 10.1080/08989621.2023.2256650 37694962

[ref-4] CoARA: Agreement on reforming research assessment.2022; Retrieved November 9, 2024. Reference Source

[ref-5] Conesa Carpintero E González Ramos AM : Accelerated researchers: psychosocial risks in gendered institutions in academia. *Front Psychol.* 2018;9:1077. 10.3389/fpsyg.2018.01077 30072928 PMC6062159

[ref-6] Glerup C Davies SR Horst M : ‘Nothing really responsible goes on here’: scientists’ experience and practice of responsibility. *J Responsible Innov.* 2017;4(3):319–336. 10.1080/23299460.2017.1378462

[ref-7] Gopalakrishna G Ter Riet G Vink G : Prevalence of Questionable Research Practices, research misconduct and their potential explanatory factors: a survey among academic researchers in the Netherlands. *PLoS One.* 2022;17(2): e0263023. 10.1371/journal.pone.0263023 35171921 PMC8849616

[ref-8] Haven TL Tijdink JK Martinson BC : Perceptions of research integrity climate differ between academic ranks and disciplinary fields: results from a survey among academic researchers in Amsterdam. *PLoS One.* 2019;14(1): e0210599. 10.1371/journal.pone.0210599 30657778 PMC6338411

[ref-9] Helgesson G Juth N Schneider J : Misuse of coauthorship in medical theses in Sweden. *J Empir Res Hum Res Ethics.* 2018;13(4):402–411. 10.1177/1556264618784206 29985088

[ref-10] Horbach SPJM Breit E Halffman W : On the willingness to report and the consequences of reporting research misconduct: the role of power relations. *Sci Eng Ethics.* 2020;26(3):1595–1623. 10.1007/s11948-020-00202-8 32103454 PMC7286863

[ref-11] Ioannidis JP Collins TA Baas J : Evolving patterns of extreme publishing behavior across science. *Scientometrics.* 2024;129:5783–5796. 10.1007/s11192-024-05117-w

[ref-12] Keulemans M : Wereldwijde blunder van het prinses Máxima centrum: opeens verspreidde het corona-desinformatie. Wat ging er mis? *de Volkskrant.* 26 November, 2024. Reference Source

[ref-13] Kozlov M : What the Stanford president's resignation can teach lab leaders. *Nature.* 2023. 10.1038/d41586-023-02438-3 37501000

[ref-19] Labib K Evans N Pizzolato D : Co-creating research integrity education guidelines for research institutions. *Sci Eng Ethics.* 2023;29(4): 28. 10.1007/s11948-023-00444-2 37470823 PMC10359202

[ref-20] Lagendijk A Wiering M : Practices and power in processes of transformative change: the example of “recognition and rewards” at Dutch universities. In: F. Landau-Donnelly, H. Carlsson, & A. Lagendijk (Eds.), *Reflecting on practices: New directions for spatial theories. *Agenda Publishing Limited,2024;41–66. Reference Source

[ref-21] Lasser J Bultema L Jahn A : Power abuse and anonymous accusations in academia–Perspectives from early career researchers and recommendations for improvement. *Beiträge zur Hochschulforschung.* 2021;43(1–2):48–61. Reference Source

[ref-14] Mostert S Hoogland M Huibers M : Excess mortality across countries in the Western World since the COVID-19 pandemic: â Our World in Dataâ estimates of January 2020 to December 2022. *BMJ Public Health.* 2024;2(1): e000282. 10.1136/bmjph-2023-000282 PMC13482250 42614536

[ref-22] Pizzolato D Dierickx K : Reverse mentoring to enhance research integrity climate. *BMC Res Notes.* 2022;15(1): 209. 10.1186/s13104-022-06098-w 35715865 PMC9205068

[ref-15] Pizzolato D Dierickx K : The mentor’s role in fostering research integrity standards among new generations of researchers: a review of empirical studies. *Sci Eng Ethics.* 2023;29(3): 19. 10.1007/s11948-023-00439-z 37160826

[ref-23] Pizzolato D Simm K Lofstrom E : Report on supplementary trainer guide and new training materials on RE/RI. 2024. 10.5281/zenodo.14281486

[ref-16] Stengers I : Another science is possible: a manifesto for slow science. (S. Muecke, Trans.). Polity Press,2018. Reference Source

[ref-17] Wäscher S Biller-Andorno N Deplazes-Zemp A : “I don’t want to do anything bad.” Perspectives on scientific responsibility: results from a qualitative interview study with senior scientists. *Nanoethics.* 2020;14(2):135–153. 10.1007/s11569-020-00365-5

