# Supplementary material for: The role of senior researchers in promoting good science: Obstacles and enablers
Source: Open Res Eur. 2025 Apr 24;5:21. Originally published 2025 Jan 23. [Version 2] doi: 10.12688/openreseurope.19140.2 (PMC12498318; doi:10.12688/openreseurope.19140.2)
Supplement: Peer review history

# Reviewer report: Reviewer response for version 2

**DOI:** 10.21956/openreseurope.21816.r53534

**Published:** 2025-05-10

**Authors:** Johannes JM van Delden

**Affiliations:** 1 University Medical Center Utrecht, Utrecht, The Netherlands

I would like to thank the authors for their revisions, which have certainly improved the article

Is the argument persuasive and supported by appropriate evidence?

Partly

Is the work clearly and cogently presented?

Yes

Is the topic of the essay discussed accurately in the context of the current literature?

Yes

Does the essay contribute to the cultural, historical, social understanding of the field?

Yes

Reviewer Expertise:

Medical Ethics

I confirm that I have read this submission and believe that I have an appropriate level of expertise to confirm that it is of an acceptable scientific standard.

# Reviewer report: Reviewer response for version 2

**DOI:** 10.21956/openreseurope.21816.r53593

**Published:** 2025-05-07

**Authors:** Elisa Reverman

**Affiliations:** 1 Cleveland Clinic Center for Bioethics, Cleveland, USA

Overall, this paper aims to contribute to an important topic. However, it struggles in 1) linking together disparate claims regarding issues with senior researchers, 2) mentorship and supervision, 3) integrating the insights from the chosen case studies to make a broader claim regarding the promotion of good science, and 4) making well-supported or thoroughly argued original claims. 

 On the first point, the authors often fail to build a strong and necessary bridge between claims. For instance, the authors introduce a case of misconduct with the publication on covid vaccination followed by claims related to insufficient supervision, the presence of recklessness, and accusations of power abuse. Within this section, substantially more discussion of the specific case is needed to show the presence and significance of insufficient supervision, recklessness, or power abuse. Without deeper connections being drawn here, the case doesn't do sufficient work for the authors. The following section begins with a discussion of senior researchers needing to recognize and understand their obligations, but the article doesn't previously argue that a lack of recognition or understanding is actually at the root of the issue. These are some instances of the underexplored or under-argued claims made in the paper. 

 On the second point, the authors take the time to distinguish mentorship from supervision early on in the paper, but the significance of this distinction isn't brought up again at any point. Further, the authors don't contribute any significant new ideas specific to instances where senior researchers fail to provide adequate supervision or mentorship and the downstream effects of this. While they do engage some of the relevant research on this topic, the authors themselves don't seem to make contributions that haven't already been made elsewhere on this topic. 

 On the third point, there is a significant disconnect between the case study on Covid vaccinations and the claims that it is supposed to support. The case, as written, details an instance where a junior (?) researcher who receives feedback from their supervisor on their seemingly questionable work, but proceeds to publish anyways with both themself and their supervisor as authors. The article then follows this case with a discussion of insufficient supervision, recklessness, and power abuse. However, as written, the case doesn't actually suggest a lack of supervision. In fact, the case seems to illustrate a case where a junior researcher was given appropriate feedback. The fact that they proceeded to publish anyways with their academic affiliation and with their supervisor as co-author is critically underexplained. Without more detail, it is unclear to what degree which party may or may not have participated in "recklessness" or power abuse. In some ways, the case describes a rogue post-doc who against best practices *despite* being advised not to proceed. 

 Finally, while the paper does engage with some relevant research to support the broader argument, the paper simply doesn't pose substantively original claims or arguments. The substantive evidence or support for their claims comes from other research, and the conclusions drawn have been discussed elsewhere in the literature. 

 While this paper as a whole addresses an important topic, it's disparately made claims and thin arguments lack the necessary substance to make a contribution to the literature on research integrity.

Is the argument persuasive and supported by appropriate evidence?

No

Is the work clearly and cogently presented?

Partly

Is the topic of the essay discussed accurately in the context of the current literature?

Partly

Does the essay contribute to the cultural, historical, social understanding of the field?

No

Reviewer Expertise:

research misconduct, transparency and research, clinical ethics, research ethics

I confirm that I have read this submission and believe that I have an appropriate level of expertise to state that I do not consider it to be of an acceptable scientific standard, for reasons outlined above.

# Reviewer report: Reviewer response for version 1

**DOI:** 10.21956/openreseurope.20711.r51234

**Published:** 2025-03-03

**Authors:** Simon E Kolstoe

**Affiliations:** 1 University of Portsmouth, Portsmouth, England, UK

Senior researchers are responsible for Questionable Research Practices, but not always to blame.

 I strongly agree with Susanne van den Hooff and Signe Mezinska that senior researchers play a critical role in fostering ethical research practices especially among their teams and those they are mentoring. Research is not an activity that can be learned from a text book, and as such all researchers learn by doing, and copying, the behaviours of their mentors. The senior researcher role is therefore the single most important role for promoting reliable, high quality and ethical research.

 Even so, and as van den Hooff & Mezinska rightly state, the "publish or perish" culture creates significant pressure on senior researchers to either cut corners perhaps through poor supervision, or at worst actively cheat so as to game the system and ensure their own personal success (or maybe survival in some cases). These perverse incentives are undoubtedly a major cause of concern, and also reason for the ever increasing problem of questionable research practices (QRPs). It is therefore critical that researchers are aware of documents such as the ALLEA code, and are encouraged to abide by its principles (alongside other documents such as the Declaration of Helsinki).

 In a recent survey of QRPs reported in https://tinyurl.com/yay8sa3m it was found that the requirement to publish regularly led to high pressure environments that subsequently made mistakes more likely. It might therefore be concluded that the majority of QRPs are not necessarily deliberate, but rather a consequence of high pressure environments, inadequate quality control, and thus potentially poor supervision or oversight from senior researchers. The results of the QRP survey seem to match the discussion in this article.

 Where inadequate supervision crosses over into exploitation of junior researchers is a related issue. If the pressure is to get results, and junior researchers are struggling to do this, it is not suprising that tensions, and potentially bullying, occur. Furthermore, power imbalances make it hard for junior researchers to take action, or defend themselves, when so much of their career success relies on their supervisors or group leaders. van den Hooff and Mezinska are therefore again accurate in emphasising this problem.

 Given the above, the question at the beginning of the conclusion to the article " *How can the academic community and policy makers support senior researchers to be good supervisors, team managers and to ethically fulfil their responsibilities?*" is highly relevant.

 The authors give two solutions - a " *slow science approach with a focus on quality over quantity*", and second " *more training courses specifically tailored to the unique challenges and responsibilities of senior researchers*". It is hard to disagree with these solutions, but at the same time hard to see how they can be implemented in the current research environment. To implement these a further step back is needed to the level of research funding, wherein such solutions become an expectation of funding, alongside the expectation of publication. Indeed until "slow science" and "researcher development" are rewarded equally to research publication, it is difficult to see how the current situation will change.

 I commend the authors on this thoughtful essay as any effort to publicise these issues is welcome. But it is also important that these topics (and indeed essays) are brought to the attention of those who hold the purse strings of research if any change is to occur.

Is the argument persuasive and supported by appropriate evidence?

Yes

Is the work clearly and cogently presented?

Yes

Is the topic of the essay discussed accurately in the context of the current literature?

Yes

Does the essay contribute to the cultural, historical, social understanding of the field?

Partly

Reviewer Expertise:

Research Ethics

I confirm that I have read this submission and believe that I have an appropriate level of expertise to confirm that it is of an acceptable scientific standard.

# Response

**Published:** 2025-04-07

**Authors:** Signe Mežinska

**Affiliations:** Institute of Clinical and Preventive Medicine, University of Latvia Faculty of Medicine and Life Sciences, Jelgavas Str. 3, Riga, Latvia

Dear Prof. Kolstoe, Thank you for your insightful and positive review! The role of senior researchers in fostering ethical research practices is indeed critical, especially given the pressures of the ‘publish or perish’ culture. Implementing solutions such as the slow science approach and tailored training courses will require significant changes in the academic culture and research funding landscape. Your support and recognition of these efforts are greatly appreciated.

# Reviewer report: Reviewer response for version 1

**DOI:** 10.21956/openreseurope.20711.r50902

**Published:** 2025-02-25

**Authors:** Johannes JM van Delden

**Affiliations:** 1 University Medical Center Utrecht, Utrecht, The Netherlands

This is a fine article, that addresses a pervasive problem in science. It is disappointing that a decade after the Lancet published its series on research waste, the scientific community still has to be reminded of the detrimental effects of a publish or perish culture. Not only detrimental for the scientists but also for society, which keeps paying for the production of too many articles too much of which is still research waste.

 The article contributes to the awareness of the problem and unfortunately that is still necessary to do. It nicely describes how publish or perish leads to increased pressure which comes with power misuse and reluctance to report misconduct.

 The article could win in influence if more text was written about the enablers of good science. That part is now restricted to the conclusion, but given the title, I would have expected more indications of how to promote good science. Currently the authors advocate slow science (please elaborate!), the recognition of diverse outputs and training courses. But I think more should be added here. In my institution for instance the matrix to evaluate researchers was radically changed from looking at H-indices only to the TRIPLE system, which looks at Team spirit, Research output, societal Impact, professional performance, Leadership and Education. By valuing all these areas researchers get the clear message that publishing is not the only way to be a good scientist.

Is the argument persuasive and supported by appropriate evidence?

Partly

Is the work clearly and cogently presented?

Yes

Is the topic of the essay discussed accurately in the context of the current literature?

Yes

Does the essay contribute to the cultural, historical, social understanding of the field?

Yes

Reviewer Expertise:

Medical Ethics

I confirm that I have read this submission and believe that I have an appropriate level of expertise to confirm that it is of an acceptable scientific standard, however I have significant reservations, as outlined above.

# Response

**Published:** 2025-04-07

**Authors:** Signe Mežinska

**Affiliations:** Institute of Clinical and Preventive Medicine, University of Latvia Faculty of Medicine and Life Sciences, Jelgavas Str. 3, Riga, Latvia

Dear Prof. van Delden,

 Thank you for your thoughtful and constructive feedback.

 We appreciate your positive evaluation of the manuscript and thank you for your suggestions for improvement of the paper, particularly regarding the expanding information on enablers of good science. We have addressed these suggestions by expanding the “Discussion and conclusions” part and including examples of good practices.

 Thank you once again for your insightful comments and support!

# Reviewer report: Reviewer response for version 1

**DOI:** 10.21956/openreseurope.20711.r50569

**Published:** 2025-02-07

**Authors:** Mohammad Hosseini

**Affiliations:** 1 Northwestern University, Evanston, Illinois, USA

This paper aims to provide a perspective on the role of senior researchers in promoting good science. ****The topic is interesting and relevant, but the analysis lacks depth or structure. Particularly, the lack of any structure is noticeable, since the paper moves rather haphazardly from one topic to the next. I suggest using a framework or some kind of structure to make the paper more readable.

 Furthermore, the term “senior researcher” is vaguely defined as “those who oversee a research group”. I have seen PhD or postdocs who oversee the work of a few graduate students, but that doesn't make them senior. Groups can be anything from 2 researchers to hundreds of researchers and overseeing these could be very different. Depending on the domain, the role of supervisors can be very different as well, think, for example, about the difference between a supervisor in humanities versus a supervisor in ecology and conservation where most of the research happens outdoors. Another suggestion is to make a distinction between supervision and mentorship (and staying away from the latter to ensure focus). The use of the term “ethical responsibilities” is confusing.

 While the paper uses statements from the ALLEA code of conduct (aimed for researchers in the EU) to highlight senior researchers’ responsibilities, it remains unclear whether ALLEA (or any of the cited papers) use the same definition for “senior researcher”.

 The paper also uses a 2024 case in the Netherlands that “drew widespread media attention to a professor accused of gross negligence” but offers no additional insights about what should have happened to the postdoc or their supervisor and kind of brushes everything off and notes that senior researchers are vulnerable like juniors and then goes on to some other examples. Please see attached file here- *https://s3-eu-west-1.amazonaws.com/openreseurope/linked/246028.reviewed_file.pdf*

Is the argument persuasive and supported by appropriate evidence?

Partly

Is the work clearly and cogently presented?

Partly

Is the topic of the essay discussed accurately in the context of the current literature?

Partly

Does the essay contribute to the cultural, historical, social understanding of the field?

No

Reviewer Expertise:

Research ethics and integrity, AI Ethics, Open Science

I confirm that I have read this submission and believe that I have an appropriate level of expertise to state that I do not consider it to be of an acceptable scientific standard, for reasons outlined above.

# Response

**Published:** 2025-04-07

**Authors:** Signe Mežinska

**Affiliations:** Institute of Clinical and Preventive Medicine, University of Latvia Faculty of Medicine and Life Sciences, Jelgavas Str. 3, Riga, Latvia

Dear Prof. Husseini, thank you for your detailed and insightful comments!

 They are very valuable for enhancing our manuscript. We are giving our responses to your comments below.

***Reviewer Comment: **"This paper aims to provide a perspective on the role of senior researchers in promoting good science. ****The topic is interesting and relevant, but the analysis lacks depth or structure. Particularly, the lack of any structure is noticeable, since the paper moves rather haphazardly from one topic to the next. I suggest using a framework or some kind of structure to make the paper more readable."*

**Author Response: **Thank you for your valuable feedback. We appreciate your suggestion and have incorporated a more structured approach by changing some parts of the manuscript to enhance the readability and depth of our analysis.

***Reviewer Comment: **"Furthermore, the term “senior researcher” is vaguely defined as “those who oversee a research group”. I have seen PhD or postdocs who oversee the work of a few graduate students, but that doesn't make them senior. Groups can be anything from 2 researchers to hundreds of researchers and overseeing these could be very different. Depending on the domain, the role of supervisors can be very different as well, think, for example, about the difference between a supervisor in humanities versus a supervisor in ecology and conservation where most of the research happens outdoors. [..] While the paper uses statements from the ALLEA code of conduct (aimed for researchers in the EU) to highlight senior researchers’ responsibilities, it remains unclear whether ALLEA (or any of the cited papers) use the same definition for “senior researcher”."*

**Author Response: **We appreciate your concern regarding the definition of senior researcher. In the amended manuscript, we have clarified the definition of senior researchers as researchers with extensive experience and expertise who oversee and supervise a research group. While we acknowledge that the use of this term can vary by field and region, we believe it is still meaningful to discuss the role of senior researchers in fostering research integrity based on the definition we suggest. Our definition aligns with the broad use of this term in the ALLEA European Code of Conduct for Research Integrity. Additionally, we note that no unified definition of senior researcher is available in the literature. We hope this clarification addresses your concerns.

***Reviewer Comment: **"Another suggestion is to make a distinction between supervision and mentorship (and staying away from the latter to ensure focus)."*

**Author Response: **Thank you for this suggestion! We have explained the difference between supervision and mentorship and made necessary changes.

***Reviewer Comment: **"The use of the term “ethical responsibilities” is confusing."*

**Author Response: **Thank you for your feedback regarding the use of the term "ethical responsibilities." We agree that this term may have caused some confusion. To clarify, we have revised the manuscript and changed the terminology.

***Reviewer Comment: **"The paper also uses a 2024 case in the Netherlands that “drew widespread media attention to a professor accused of gross negligence” but offers no additional insights about what should have happened to the postdoc or their supervisor and kind of brushes everything off and notes that senior researchers are vulnerable like juniors and then goes on to some other examples."*

**Author Response: **Thank you for your feedback regarding this case! We appreciate your concern about the depth of analysis provided. However, we believe that an in-depth analysis of this specific case is not necessary for the scope of this paper. The references we have included offer readers the opportunity to explore the case further and gain more detailed insights. Our intention was to highlight the broader issue of vulnerability among senior researchers, similar to early career researchers, and to provide a range of examples to support this discussion. We hope this approach aligns with the objectives of our paper and provides sufficient context for our readers.
